# Effect of both protective and reducing agents in the synthesis of multicolor silver nanoparticles

**DOI:** 10.1186/1556-276X-8-101

**Published:** 2013-02-22

**Authors:** Pedro Jose Rivero, Javier Goicoechea, Aitor Urrutia, Francisco Javier Arregui

**Affiliations:** 1 Nanostructured Optical Devices Laboratory, Electric and Electronic Engineering Department, Public University of Navarra, Edif. Los Tejos, Campus Arrosadía, Pamplona 31006, Spain

**Keywords:** Silver nanoparticles, Multicolor, Morphology, Localized surface plasmon resonance

## Abstract

In this paper, the influence of variable molar ratios between reducing and loading agents (1:100, 1:50, 1:20, 1:10, 1:5, 1:2, 1:1, 2:1) and between protective and loading agents (0.3:1, 0.75:1, 1.5:1, 3:1, 7.5:1, 30:1, 75:1) in the synthesis of silver nanoparticles by chemical reduction has been evaluated to obtain multicolor nanoparticles with a high stability in time. The protective agent poly(acrylic acid, sodium salt) (PAA) and reducing agent dimethylaminoborane (DMAB) play a key role in the formation of the resultant color. Evolution of the optical absorption bands of the silver nanoparticles as a function of PAA and DMAB molar ratios made it possible to confirm the presence of silver nanoparticles or clusters with a specific shape. The results reveal that a wide range of colors (violet, blue, green, brown, yellow, red, orange), sizes (from nanometer to micrometer), and shapes (cubic, rod, triangle, hexagonal, spherical) can be perfectly tuned by means of a fine control of the PAA and DMAB molar concentrations.

## Background

Metal nanoparticles (NPs) (e.g., Ag, Au, Cu NPs) have attracted great interest in a number of disciplines because of their potential applications in optical, medical, or electronic devices. The control of their size and shape is a challenging goal, and a large number of reports have been published for the preparation of metal nanoparticles of various morphologies [1-5], mainly for plasmonic and sensing applications [6]. Very recently, our group has incorporated silver nanoparticles (AgNPs) in polymeric films for detecting fast changes of humidity (human breathing) [7,8] and, at the same time, preventing the growth of bacteria very likely in high-humidity atmosphere [9-11].

One of the most frequently used methods is the production of AgNPs from aqueous solutions of Ag+ salts by exposure to radiation (ambient light, UV–vis, gamma) [12-15] or via chemical reduction [16,17]. A wide number of solvents and encapsulating agents have been used to produce AgNPs and prevent their agglomeration [18-21]. However, the addition of water-soluble polymers such as poly(acrylic acid, sodium salt) (PAA) made possible a better control of the particle growth. This polymer in aqueous solution produces polyacrylate anions (PA^−^) with uncoordinated carboxylate groups which can bind metallic cations such as silver (Ag+ salts), forming intermediate charged clusters [22,23]. Due to this, PAA is of special interest because it can control and stabilize both silver nanoparticles and clusters along the polymeric chains with a high stability in time. Several groups of investigation have carried out experiments to report the composition and evolution of these positively charged clusters [24-26].

One of the most relevant aspects of the synthesis of AgNPs is that their optical properties (the resultant color) present high dependence on their crystal morphology (specially size and shape) [27,28]. These AgNPs exhibit localized surface plasmon resonance (LSPR) spectra (colors), enabling the monitoring of their evolution and color formation by UV–vis measurements.

In this work, the aim is the development of an easy chemical method to synthesize both clusters and silver nanoparticles of different colors in aqueous polymeric solution at room temperature and in a short period of time with a well-defined shape, using PAA as protecting agent. With this goal, an experimental matrix of results is generated by changing two parameters: the concentration of the protecting agent PAA (from 1 to 250 mM); and the different molar ratio between the reducing agent, dimethylaminoborane (DMAB) (concentration from 0.033 to 6.66 mM), and the loading agent, silver nitrate (AgNO_3_) (at a fixed concentration of 3.33 mM). The experimental matrix is formed by 56 different combinations of protective agent concentration and ‘reducing agent/loading agent’ ratio. From these 56 combinations, a wide range of AgNPs can be obtained with different colors (yellow, orange, red, violet, blue, green, brown) and tunable shape and size. Henceforward, for the sake of simplicity, this experimental matrix will be named the multicolor silver map. To our knowledge, this is the first time that an experimental study based on the influence of both PAA and DMAB molar concentrations to obtain colored silver nanoparticles and clusters has been reported in the literature.

## Methods

### Materials

The materials used were as follows: poly(acrylic acid, sodium salt) 35 wt.% solution in water (Mw 15.000), silver nitrate (>99% titration), and dimethylaminoborane complex. All chemicals were purchased from Sigma-Aldrich Corporation (St. Louis, MO, USA) and used without any further purification. All aqueous solutions were prepared using ultrapure water with a resistivity of 18.2 MΩ·cm.

### Preparation of the multicolor silver map

A chemical reduction method at room temperature was performed using AgNO_3_ as loading agent, DMAB as reducing agent, and PAA as protective agent. In order to investigate the influence of both PAA and DMAB on color formation, several concentrations of this water-soluble polymer (from 1 to 250 mM PAA) and reducing agent (from 0.033 to 6.66 mM DMAB) were prepared. The samples of the multicolor silver map have been synthesized several times under the same experimental conditions (room conditions), and no significant difference in the optical absorption spectra of the AgNPs was observed.

### Characterization

Transmission electron microscopy (TEM) was used to determine the morphology of both silver nanoparticles and clusters. TEM analysis was carried out with a Carl Zeiss Libra 120 (Carl Zeiss, AG, Oberkochen, Germany). Samples for TEM were prepared by dropping and evaporating the solutions onto a collodion-coated copper grid.

UV-visible (vis) spectroscopy was used to characterize the optical properties of the multicolor silver map. Measurements were carried out with a Jasco V-630 spectrophotometer (Jasco Analytical Instruments, Easton, MD, USA).

## Results and discussion

### Multicolor silver map

**Figure 1:**
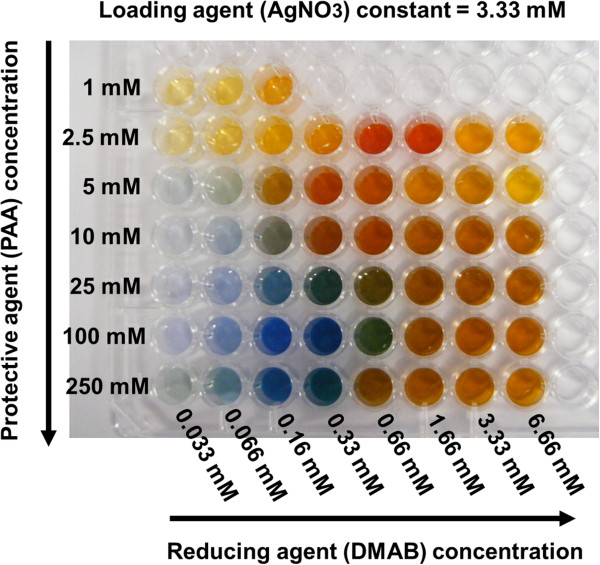
**Photograph of multicolor silver map obtained as function of variable protective (PAA) and reducing (DMAB) agents.**

The samples were prepared by adding freshly variable DMAB concentrations (0.033, 0.066, 0.16, 0.33, 0.66, 1.66, 3.33, and 6.66 mM) to vigorously stirred solutions which contained different PAA concentrations (1.0, 2.5, 5.0, 10.0, 25.0, 100.0, and 250.0 mM) and to a constant AgNO_3_ concentration (3.33 mM). The final molar ratios between the reducing and loading agents (DMAB/AgNO_3_ ratio) were 1:100, 1:50, 1:20, 1:10, 1:5, 1:2, 1:1, and 2:1. The final molar ratios between the protective and loading agents (PAA/AgNO_3_ ratio) were 0.3:1, 0.75:1, 1.5:1, 3:1, 7.5:1, 30:1, and 75:1. Once the reaction was completed, the color was stable without any further modification. In Figure 1, it is possible to appreciate the wide range of colors (yellow, orange, red, violet, blue, green, or brown) which were obtained when both PAA and DMAB molar concentrations were varied. The synthesized AgNP dispersions showed no changes in the position of their optical absorption bands even after 6 months of storage at room conditions.

### Effect of the protective agent

One of the major findings of the present study was the significant influence of the PAA concentration on the final color of each sample. Due to its molecular structure with PA^−^ in water solution, the binding of PA^−^ with metal cations (silver) was made possible, forming Ag^+^PA^−^ complexes wherein a posterior reduction of the silver cations to silver nanoparticles takes place [24-26]. Moreover, PAA concentration plays a key role for the stabilization of silver nanoparticles and metal clusters along the polymeric chains, controlling their size and shape. In fact, the multicolor silver map of Figure 1 demonstrates that with a lower PAA concentration (1 or 2.5 mM), stable silver nanoparticles are generated, showing only yellow, orange, and red colors. These AgNPs showed no changes in the position of their optical absorption bands even after 6 months. Our study demonstrates that by increasing the PAA concentration from 5 to 250 mM, a wider range of colors (violet, blue, green, brown, orange) is obtained with a high stability in time. In fact, a higher range of blue colors is obtained for higher PAA concentrations (25, 100, or 250 mM; see Figure 1). This blue color has been reported in previous works using photochemical or chemical reduction [14,15,17], but not using DMAB as reducing agent in the presence of various PAA concentrations.

**Figure 2:**
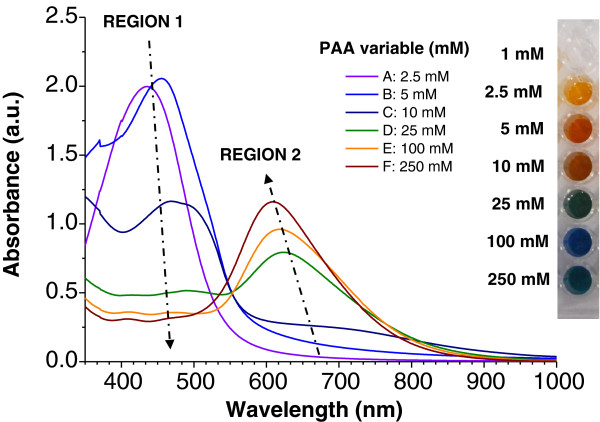
**UV**–**vis absorption spectra of silver solutions at a constant DMAB concentration. **They are prepared with different PAA concentrations at a constant DMAB concentration of 0.33 mM (fourth column of the silver multicolor map of Figure 1).

Figure 2 shows the UV–vis spectra for different PAA concentrations, from 2.5 to 250 mM, when the DMAB concentration was kept constant (0.33 mM); this can be seen in the fourth column of Figure 1. It is important to remark that 1 mM PAA for this DMAB concentration or higher DMAB concentration produces a complete precipitation of silver, and no color formation is obtained. The UV–vis spectra reveal the evolution of two spectral regions (region 1 for the 400- to 500-nm band and region 2 for the 600- to 700-nm band) as a function of PAA concentration. Initially, according to the yellow and orange colors obtained for the lower PAA concentrations of 2.5 and 5 mM, an intense absorption band is obtained at short wavelengths with the wavelength of maximum absorbance located at 435 and 445 nm, respectively (region 1). As the PAA concentration is increased (10 mM), the absorption band in region 1 decreases in intensity and shifts to longer wavelengths with a change in the resulting color (brown, 10 mM); at the same time, a new absorption band appears in region 2 (600 to 700 nm), indicating the synthesis of silver nanoparticles of different shapes as compared with those seen in previously obtained colors with lower PAA concentration. In addition, when the PAA molar concentration is increased from 25 to 250 mM, the generation of new colors is achieved (blue or green) with an increase in the intensity of their absorption bands in region 2, whereas simultaneously, a gradual decrease in intensity in region 1 is observed.

**Figure 3:**
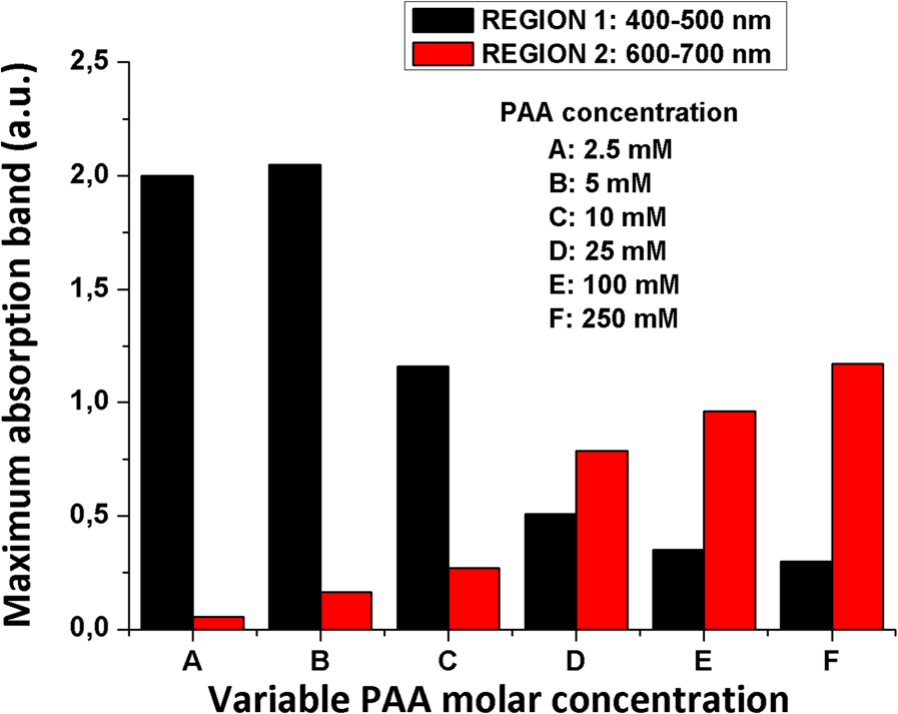
**Evolution of UV**–**vis maxima absorption bands of the silver sols in regions 1 and 2. **Absorption bands in regions 1 and 2 are 400 to 500 nm and 600 to 700 nm, respectively. They are prepared with different PAA concentrations at a constant molar DMAB concentration (0.33 mM).

Figure 3 was also plotted in order to show a clearer picture of the evolution of optical absorption bands (regions 1 and 2) when the concentration of PAA was increased. As can be deduced from Figure 3, PAA plays a key role in the formation of the resulting color because well-defined positions of the maximum absorption bands as a function of PAA concentration added to the solution are clearly observed. These changes in color from orange (lower PAA concentration with an intense absorption band in region 1) to blue (higher PAA concentration with an absorption band in region 2) can be perfectly controlled during the synthesis process as a function of PAA and DMAB added in the initial solution.

**Figure 4:**
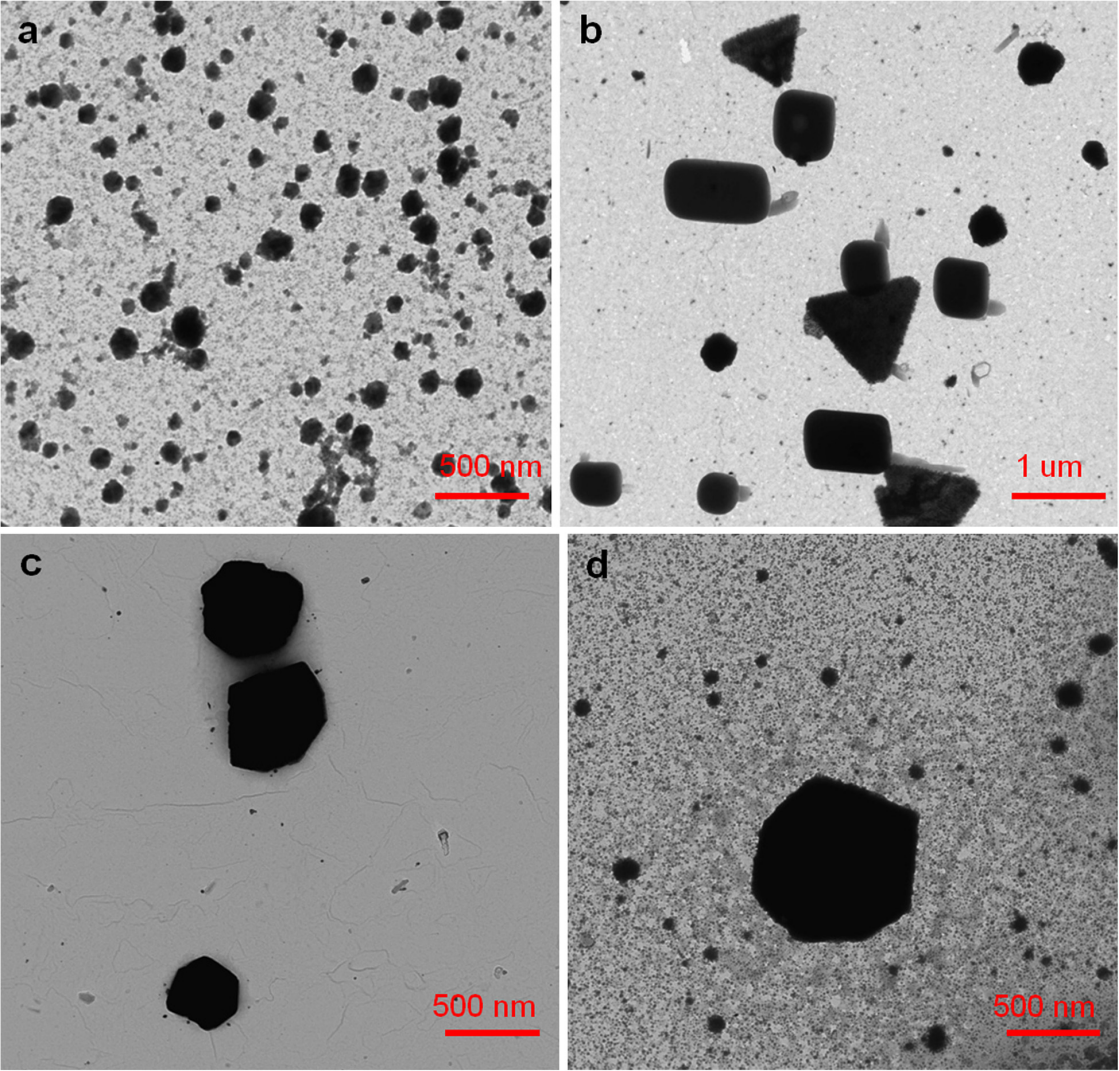
**TEM micrographs that show the formation of AgNPs with different shapes for different PAA concentrations. **(**a**) Spherical shape for 2.5 mM PAA. (**b**) Several shapes (triangle, rod, cube, bar) for 10 mM PAA. (**c**, **d**) Hexagonal shapes for 100 and 250 mM PAA, respectively. The DMAB concentration was 0.33 mM.

In the opinion of the authors, the reason for the gradual absence of the plasmonic resonance band in region 1 (around 410 nm) for higher PAA concentrations is due to the gradual absence of silver nanoparticles with spherical shape and the gradual appearance of silver nanoparticles with new shapes. This hypothesis is corroborated by the results obtained by TEM. As can be seen in Figure 4, PAA concentrations from 5 to 250 mM led to the formation of new shapes (rods, cylinders, triangles, cubic, hexagon) with a considerable increase in size with respect to the AgNPs obtained with lower PAA concentrations (1 or 2.5 mM) where only spherical shapes were observed.

The results reveal that varying the PAA concentration induces a change in the shape and size of the particles from 100 to 300 nm (nanoparticles) with lower PAA concentration (orange color) to 0.5 to 1 μm (clusters) with higher PAA concentration (brown, green, or blue color).

### Effect of the reducing agent

**Figure 5:**
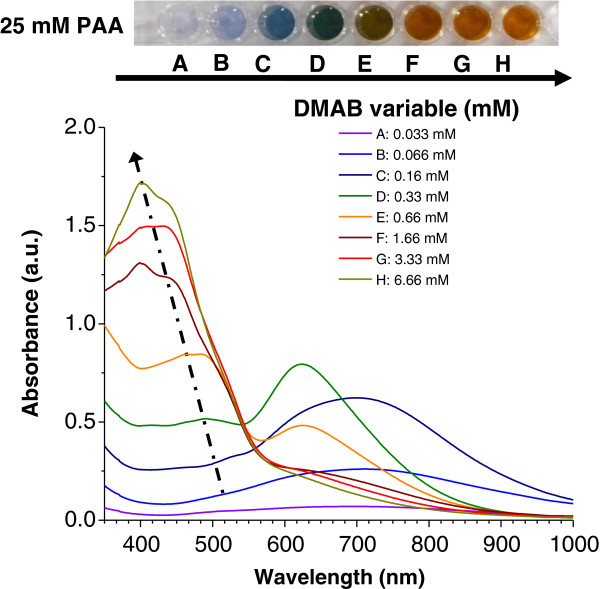
**UV**–**vis absorption spectra of silver solutions at a constant PAA concentration. **They are prepared with different DMAB concentrations at a constant PAA concentration of 25 mM (fifth line of the silver multicolor map of Figure 1).

In addition, to study the influence of the molar concentration of the protective agent (PAA), the reduction of silver cations at different reducing agent/loading agent molar ratios (DMAB/AgNO_3_ ratio) was also noted. When the reducing agent is increased from 0.033 to 6.66 mM DMAB in the same mixture of AgNO_3_ and PAA, the maximum absorption band is shifted to shorter wavelengths (region 1). Figure 5 shows the UV–vis absorption bands when the reducing agent DMAB concentration is increased in 25 mM PAA solution (fifth line in Figure 1). As can be seen in Figure 5, an increase of the reducing agent DMAB produces an absorption band shift to shorter wavelengths. An intense absorption band at 410 nm is observed when the highest DMAB proportion (6.66 mM) is added to the mixture and an orange color is obtained, indicating the synthesis of spherical AgNPs (corroborated by TEM).

The spectra reveal that the evolution of the absorption bands as a function of the DMAB added to the solution shows just the opposite behavior to the phenomenon observed when PAA was added. The position of the maximum absorption bands shifted to shorter wavelengths when DMAB concentration was increased, and the resulting colors are formed in a different order (from violet to orange) during the synthesis process.

According to the results shown in Figure 5, the evolution of both regions demonstrated that an absorption band at long wavelengths (region 2) is obtained in the first steps of color formation (violet or blue) with lower DMAB molar in the solution. However, when the DMAB molar was increased, the maximum absorption band shifted to short wavelengths (region 1) with a corresponding change of color (brown or green). Furthermore, when higher DMAB molar was added to the solution (with orange color only), a new intense absorption band appeared at 410 nm which was indicative of the formation of nanoparticles with spherical shape. These same spectral absorption variations in both regions have been observed with higher PAA concentrations (100 or 250 mM).

**Figure 6:**
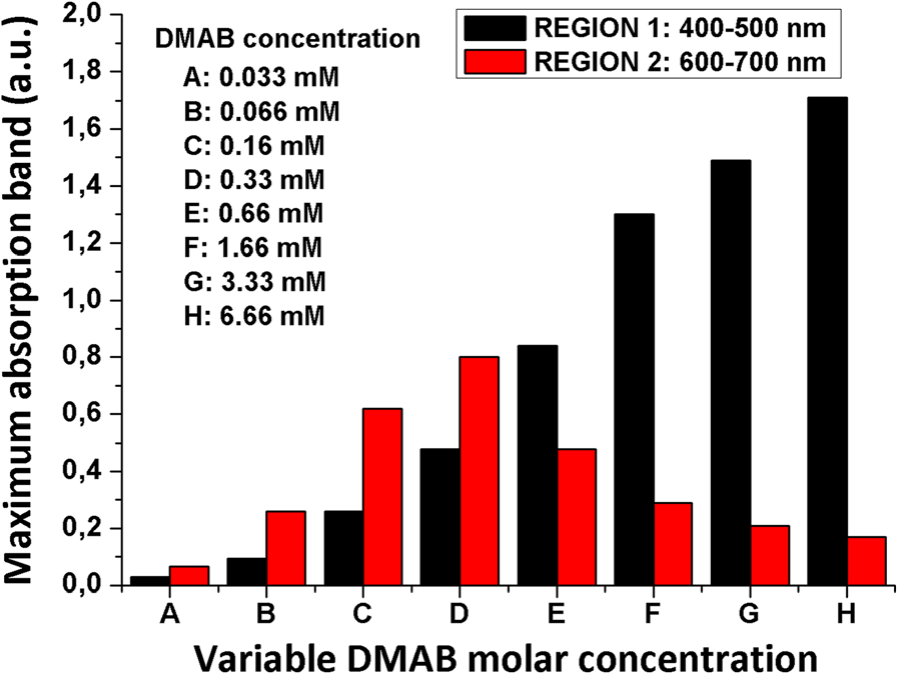
**Evolution of UV**–**vis maxima absorption bands of silver sols in regions 1 and 2. **Absorption bands in regions 1 and 2 are 400 to 500 nm and 600 to 700 nm, respectively. They are prepared with different DMAB concentrations at a constant molar PAA concentration (25 mM) and a constant molar DMAB concentration.

Similar to what was made in the preceding section, Figure 6 was also plotted in order to show a clearer picture of the evolution of the optical absorption bands (regions 1 and 2) when the concentration of DMAB was increased. In Figure 6, it is easy to identify the absorbance increase in region 2 from 0.033 to 0.33 mM DMAB. Conversely, from 0.33 to 6.66 mM DMAB, the absorbance in region 2 decreased. The absorbance of region 1 always increases with the DMAB concentration. In view of these results, the influence of the DMAB concentration in the color of the synthesized AgNPs is also clear.

**Figure 7:**
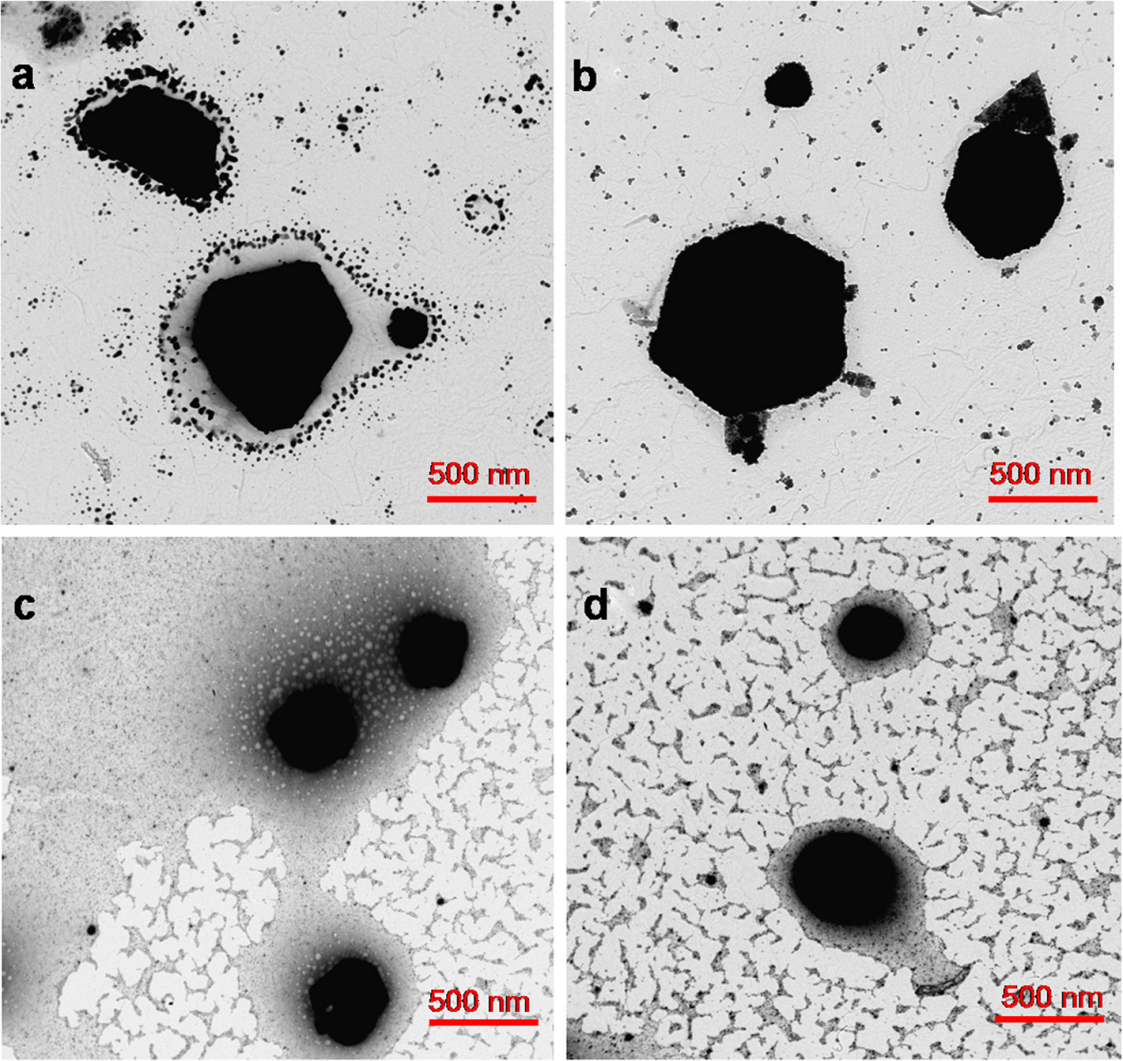
**TEM micrographs that show the formation of AgNP with different shapes for different DMAB concentrations. **(**a**) Triangle shape with 0.066 mM DMAB. (**b**) Hexagonal shape with 0.16 mM DMAB. (**c**) Quasi-spherical shape with 0.66 mM DMAB. (**d**) Spherical shape with 3.33 mM DMAB. The PAA concentration was 25 mM.

Figure 7 shows TEM micrographs of the synthesized silver nanoparticles and clusters with different DMAB molar ratios in the presence of 25 mM PAA, and in all cases, different shapes can be obtained. Initially, specific shapes (triangle or hexagonal) were obtained when lower DMAB molar (0.066 or 0.16 mM, respectively) was added (Figure 7a,b). However, these shapes and the resultant color dramatically changed (brown or orange color) when higher DMAB molar (0.66 and 3.33 mM) was added to the solution. The final position of their maximum absorption bands (UV–vis spectroscopy) was at 410 nm, and the resultant orange color indicates the excitation of the LSPR of spherical shapes (Figure 7d).

**Figure 8:**
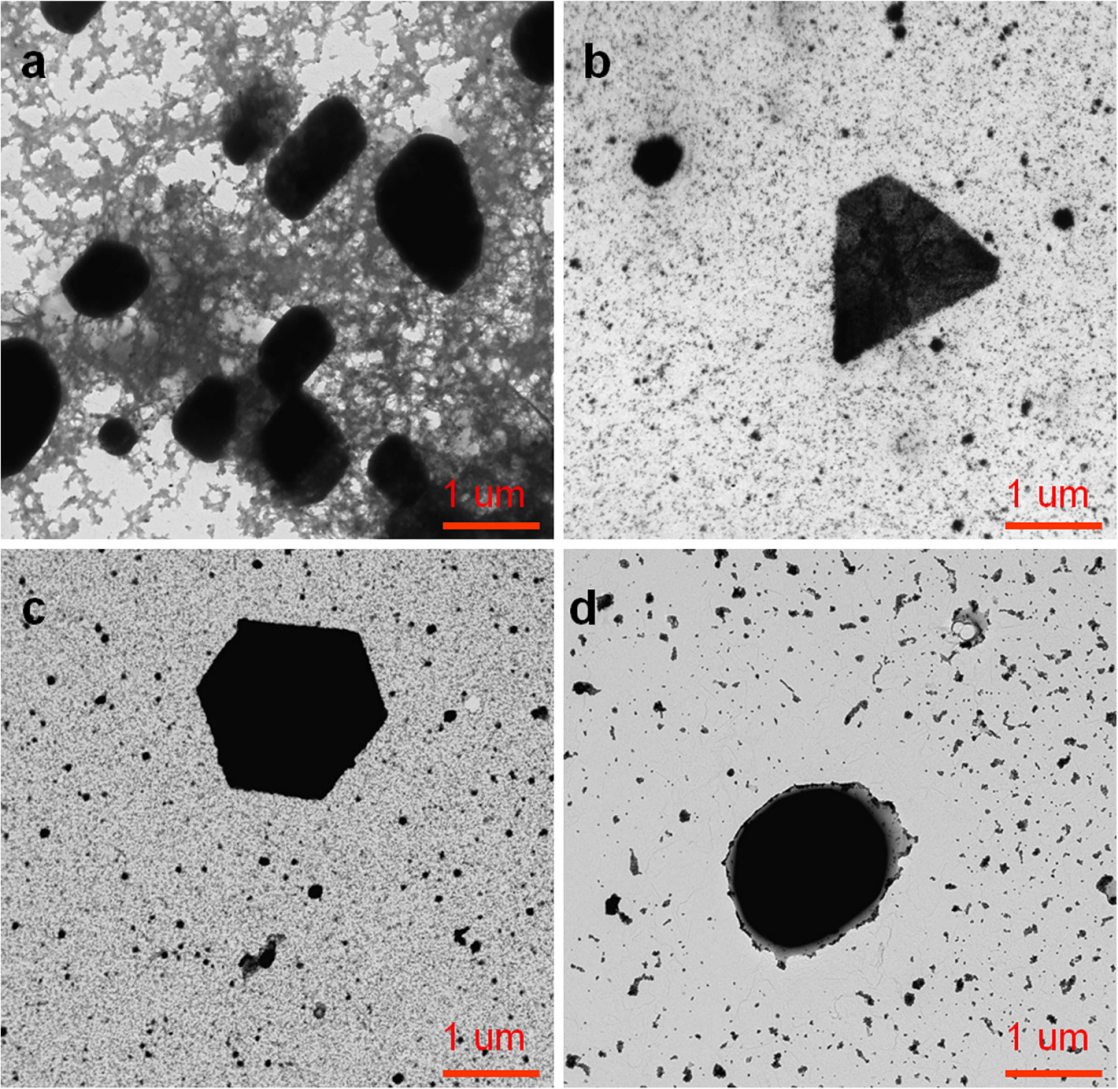
**TEM micrographs showing the formation of AgNP using 10 mM PAA and different DMAB concentrations. **(**a**) Rod shape with 0.033 mM DMAB. (**b**) Triangle shape with 0.066 mM DMAB. (**c**) Hexagonal shape with 0.66 mM DMAB. (**d**) Spherical shape with 6.66 mM DMAB.

Finally, an important aspect observed in this study is the evolution of having the same shapes (rod, triangle, hexagonal, and spherical) for different PAA concentrations when DMAB molar was gradually increased. Figure 8 shows a similar evolution in the resulting shapes as a function of DMAB molar added in the presence of 10 mM PAA. Initially, rod or triangle shapes were observed for lower DMAB molar (0.033 and 0.066 mM), but a change in the shape to hexagonal or spherical were observed when DMAB molar was increased (0.66 or 6.66 mM, respectively). In addition, UV–vis spectroscopy (not shown here) revealed identical spectral changes in the maximum absorption band in both regions. Firstly, an absorption band is obtained in region 2 that corresponds to rod, triangle, or hexagonal shapes (Figure 8a,b,c, respectively), and secondly, this absorption band was displaced to shorter wavelengths in region 1, appearing as an intense absorption band at 410 nm due to the synthesis of spherical nanoparticles (Figure 8d).

### Other considerations

**Figure 9:**
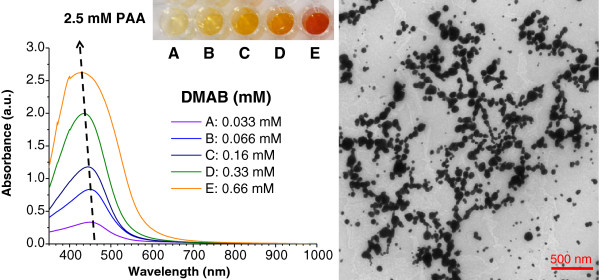
**UV**–**vis absorption spectra of silver solutions and TEM micrograph of the reddish sample. **UV–vis absorption spectra of silver solutions prepared with different DMAB concentrations at a constant PAA concentration of 2.5 mM (left), and TEM micrograph of the reddish sample (0.66 mM DMAB) with aggregation of spherical nanoparticles (right).

A relevant aspect of this work is the synthesis of silver reddish nanoparticles in the presence of 2.5 mM PAA because this color is not obtained with lower or higher PAA concentrations. In Figure 9 (left), it is possible to appreciate the evolution of the maximum absorption band (UV–vis spectroscopy) when variable DMAB molar is added to the solution. It is worth noting that the intensity of the peak corresponding to the red solution is broader than in the yellow or orange solution, indicating a considerable increase and aggregation in the number of synthesized silver nanoparticles. The maximum absorption band of this reddish solution is gradually shifted to a lower wavelength (425 nm) in comparison with orange (435 nm) or yellow (445 nm) solution. In these colors (yellow, orange, or red), the position of their maximum absorption bands in region 1 (400 to 500 nm) and the absence of absorption bands in region 2 (600 to 700 nm) indicate the complete synthesis of nanoparticles with spherical shape which is corroborated by TEM (Figure 9, right).

## Conclusions

In this study, we have successfully synthesized a multicolor silver map as a function of variable PAA and DMAB concentrations with a constant concentration of silver cations using a chemical reduction method. It has been demonstrated that a fine control of both PAA and DMAB concentrations made it possible to obtain a wide range of colors with specific shapes. Initially, only yellow, orange, or red color is obtained with lower PAA concentrations (1.0 or 2.5 mM PAA), whereas violet, blue, green, brown, or orange color is obtained with higher PAA concentrations (from 5 to 250 mM).

Samples have been characterized using TEM and UV–vis spectroscopy in order to verify the shape and evolution of their maximum absorption bands in two spectral regions (region 1, 400 to 500 nm; region 2, 600 to 700 nm). Firstly, when PAA concentration varies (from 1 to 250 mM) for a constant DMAB concentration (0.33 mM) and, secondly, when DMAB concentration varies (from 0.033 to 6.66 mM) for a constant PAA concentration (10 or 25 mM), the results indicate that for higher PAA or lower DMAB molar concentrations, an absorption band at longer wavelengths (region 2) appears, which implies violet, blue, or green solutions of AgNPs with hexagonal, triangle, and rod shapes. On the other hand, for lower PAA or higher DMAB concentrations, an intense absorption band at shorter wavelengths around 410 nm (region 1) appears, which implies orange red solutions of AgNPs of spherical shape. In summary, the fine control of PAA and DMAB concentrations in the AgNPs synthesis makes possible the color selection of the AgNPs solutions, from violet to red, as well as the shape (spherical, rod, triangle, hexagonal, cube), and size (from nanometer to micrometer) of the nanoparticles. To our knowledge, this is the first time that an experimental matrix showing multicolor silver nanoparticle solutions with well-defined shape and size using both protective agent (PAA) and reducing agent (DMAB) has been reported in the bibliography.

## Abbreviations

AgNO3: Silver nitrate; AgNPs: Silver nanoparticles; DMAB: Dimethylaminoborane; LSPR: Localized surface plasmon resonance; PAA: Poly(acrylic acid sodium salt); TEM: Transmission electron microscopy

## Competing interests

The authors declare that they have no competing interests.

## Authors’ contributions

PJR carried out the main part of the experimental work and the UV–vis measurements and TEM images. He participated in the design of the study and in the draft of the manuscript. JG participated in the experimental work, carried out the UV–vis measurements, and contributed to draft the manuscript. AU participated in the experimental work and carried out the TEM images. FJA participated in the design of the study and helped to draft the manuscript. All authors read and approved the final manuscript.
